# Patient-Rated Wrist Evaluation Threshold for Successful Open Surgery of the Triangular Fibrocartilage Complex

**DOI:** 10.1055/s-0043-1771010

**Published:** 2023-07-07

**Authors:** Reinier Feitz, Yara E. van Kooij, Mark J. W. van der Oest, J Sebastiaan Souer, Steven E. R. Hovius, Ruud W. Selles, the Hand–Wrist Study Group

**Affiliations:** 1 Department of Plastic, Reconstructive, and Hand Surgery, Erasmus MC, Rotterdam, The Netherlands; 2 Hand and Wrist Center, Xpert Clinics, Amsterdam, The Netherlands; 3 Department of Plastic, Reconstructive and Hand Surgery, Radboud University Medical Center, Radboud Institute for Health Sciences, Nijmegen, The Netherlands; 4 Department of Rehabilitation Medicine, Erasmus MC, Rotterdam, The Netherlands; 5 Xpert Clinics, Xpert Handtherapie, Flight Forum, Eindhoven, The Netherlands

**Keywords:** TFCC, triangular fibrocartilage, PROM, surgery, open reinsertion, outcome, prognostic factors

## Abstract

**Purpose** To determine thresholds in patient-reported outcome measures at baseline in patients electing to undergo triangular fibrocartilage complex (TFCC) surgery to select patients with clinically improved outcomes.

**Methods** The study cohort comprised consecutive patients who underwent open TFCC repair between December 2011 and December 2018 in various clinics in the Netherlands. All patients were asked to complete the patient-rated wrist evaluation (PRWE) questionnaire at baseline as well as at 12 months postoperatively. The minimal clinically important difference (MCID) for the PRWE was calculated to be 24 using an anchor-based method. We compared patient, disease, and surgical characteristics between patients who did and did not reach the MCID. The *t* -tests and chi-square tests were undertaken to test differences between outcomes and satisfaction in patients who did or did not reach the MCID.

**Results** Patients (34%) who did not reach MCID had a longer history of complaints. The chances of reaching the MCID for patients with a low PRWE score at baseline were slim. Of patients with a PRWE score <34 at baseline, only 14% reached the MCID, whereas in patients with a PRWE score of ≥34, 69% reached the MCID.

**Conclusion** A PRWE total score at baseline <34 is a strong signal to reconsider open surgery of the TFCC because the chance of reaching a clinically meaningful outcome is slim.

**Level of Evidence** II.

**Type of Study** Therapeutic.

As hand surgeons, we seek guidance for the indications for surgical interventions. Ideally, this requires prognostic factors that demonstrate sufficient correlation with the outcome to justify treatment selection. These factors are scarce and difficult to identify. We tend to look for patient factors such as comorbidity (e.g., diabetes mellitus, tobacco smoking) to discover a correlation that contributes to the decision-making process for elective surgery.

Studies have shown that not all patients may benefit from open triangular fibrocartilage complex surgery (TFCCS). 1 2 In our studies on outcomes after TFCCS, 3 4 we found that 20% of patients did not reach the minimal clinically important difference (MCID) of the patient-rated wrist evaluation (PRWE). That study gave insights into outcomes for open TFCCS, but it provided limited insight into which patients carry a high chance of failure to reach an MCID.

Therefore, in the present study, we asked whether a baseline patient-reported outcome measurement (PROM), such as the PRWE, could predict success or failure to reach MCID for open reinsertion of the TFCC. And, we questioned whether the patients who failed to reach the MCID were satisfied or dissatisfied with the treatment experience. We knew from our studies 5 6 7 that patient's experience and illness perception are important indicators for surgical outcome in various hand-surgery treatments. Hence, were the patients whose procedure was unsuccessful with a low PRWE score at baseline dissatisfied in general? Did they have a bad treatment experience that could contribute to a perceived worse outcome?

To answer these questions, we undertook a detailed analysis of the differences in patients who meaningfully improved and who did not after TFCCS. Moreover, we searched for a threshold value for the PRWE at baseline that could predict a clinically improved outcome.

## Methods

The ethics committee of Erasmus University Medical Center approved our study protocol (NL-sl/MEC-2018-1088). Patients provided written informed consent for their data to be used in this study. Our institution comprises 18 handsurgery centers in the Netherlands.

A retrospective review was undertaken on data of consecutive patients who elected to have open repair of the TFCC between December 2011 and December 2018. Patients were invited to be part of a routine system for outcome measurement after their first consultation with a surgeon. After providing informed consent, they received online questionnaires at baseline as well as 3 and 12 months after surgery. This routine care in our institution also included measurements of the range of movement preoperatively and at 3- and 12-month follow-ups. Three reminders were sent to patients for each round of questionnaires. Patients who failed to complete questionnaires at baseline or 12-month follow-up were excluded. The clinical and research setting of our study group has been described in more detail. 8 9

Usually, the indication for open TFCC reinsertion was a foveal tear with instability of the distal radioulnar joint (DRUJ). 10 11 Management of ulnar-sided wrist problems followed specific steps. Briefly, nonoperative treatment was initiated by short-term immobilization, followed by a rigorous program of wrist exercise, 6 to 12 weeks of supervised therapy by a hand therapist. If symptoms persisted for >3 months, DRUJ instability was evident, clinical symptoms and/or radiographs with a flake or nonunion of the ulnar styloid were present, then proceeding to direct open repair of the TFCC was considered. In all other cases, arthroscopy or magnetic resonance imaging of the wrist was done to confirm that a TFCC injury was present.

Patients received a regional local anesthetic block (axillary or supraclavicular) by anesthetists who each provides >800 upper-extremity blocks per year. Surgeons undertook their preferred method of open TFCC reinsertion. Most surgeons used a method derived from that of Garcia-Elias et al. 10 The surgical procedure and rehabilitation have been described in detail by the first author. 4 Foveal reattachment and tightening of the dorsal capsule was used for all types of TFCC tears.

Patients were asked to complete the Dutch version of PRWE at baseline as well at 3 and 12 months after surgery. 12 PRWE is a validated questionnaire comprising 15 questions: 5 questions for pain and 10 for disability. 13 All questions are answered on a scale from 0 (“no pain or dysfunction”) to 10 (“severe pain or dysfunction”). For both subscales, a score between 0 and 50 is calculated.

Sorensen et al reported an MCID for the PRWE of 14 for nonoperative treatment for hand and wrist disorders. However, they stated that this MCID cannot be generalized to patients undergoing surgical treatment. 14 Therefore, we aimed to calculate the MCID of the PRWE specifically for patients undergoing TFCC reinsertion. We used an existing database of a different cohort of 357 patients who completed the PRWE before and 12 months after TFCC reinsertion and provided data on satisfaction with treatment. We excluded patients who underwent surgery on the same hand within 12 months after open TFCC reinsertion for concomitant disorders.

For the MCID calculation for patients undergoing open surgery for the TFCC, we used the minimal important change (MIC) predict method with satisfaction with treatment results as the anchor. This approach can correct for a bias if the proportion of patients scoring to be “improved” is not equal to the proportion “not improved.” 15 16 Patients rating their satisfaction with treatment results 12 months postoperatively as “fair,” “good,” or “excellent” were considered “improved”. There was good discriminative ability (area under the curve = 0.87) for this anchor. 17 This strategy resulted in an MCID of 24 for patients undergoing TFCC reinsertion. This process is described in another study by Hoogendam et al. 18

Patients were also asked to complete a visual analog scale for pain and hand function at baseline and 3 and 12 months. Pain and function were rated on a scale from 0 (“no pain or function”) to 100 (“severe pain and no function”). Satisfaction with the result was measured at 3- and 12-month follow-ups using a single question with a five-point scale (“poor,” “fair,” “moderate,” “good,” and “excellent”). Patients were also asked to complete a questionnaire on how they experienced the delivery of care, commonly referred to as a patient-rated experience measure (PREM). This PREM questionnaire comprised 25 items that could be classified into six subdomains: “physician communication and competence” (six items); “perioperative care” (four items); “postoperative care” (four items); “general information” (two items); “treatment information” (three items); and “quality of facilities” (six items). 19 Supplementary Material S1 (online only) contains the questions used in this PREM questionnaire.

We analyzed the medical records of all included patients to extract relevant patient, disease, and surgical factors. First, we compared patient, disease, and surgical characteristics of patients who did/did not reach the MCID. Numerical data with a normal distribution were compared using the Student's *t* -test. Numerical data with a nonnormal distribution were compared using the Wilcoxon's test. The chi-square test was employed to compare categorical data. Second, we defined a threshold by visual inspection of a graph between the PRWE intake score and the chance to reach the MCID. Third, we compared PREM data between the two groups using Student's *t* -test. Finally, we analyzed satisfaction with the treatment result in relation to the PRWE at baseline and reaching the MCID.

## Results

**Fig. 1:**
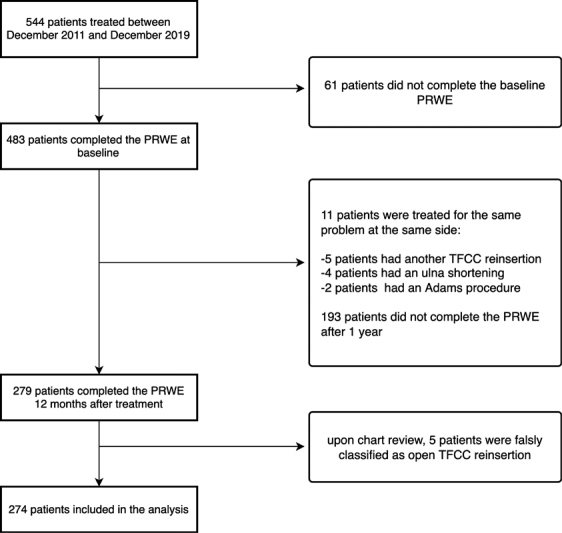
Flowchart. PRWE, patient-rated wrist evaluation; TFCC, triangular fibrocartilage complex.

Between December 2011 and December 2018, 544 patients received an open TFCC reinsertion at our centers. We excluded 61 patients due to incomplete or missing PRWE questionnaires at baseline. Another 204 patients were excluded due to incomplete or missing PRWE questionnaires 12 months after surgery, or because they underwent surgery on the same hand within 12 months after open TFCC reinsertion. In total, 274 patients were included in our study ( Fig. 1 ). All patients had a period of nonoperative management of their complaints before to the indication for surgery.

Neither the mean pronation nor supination changed significantly, from 77 degrees (standard deviation [SD] 11 degrees) at baseline to 78 degrees (SD 11 degrees) at 12 months ( *p* = 0.438, paired *t* -test), and from 75 degrees (SD 13 degrees) at baseline to 76 degrees (SD 10 degrees) at 12 months ( *p* = 0.936, paired *t* -test), respectively. Both mean wrist extension and flexion increased significantly, from 63 degrees (SD 13 degrees°) at baseline to 70 degrees (SD 11 degrees) at 12 months ( *p* < 0.001, paired *t* -test), and from 59 degrees (SD 18 degrees) at baseline to 68 degrees (SD 12 degrees) at 12 months ( *p* < 0.001, paired *t* -test), respectively. The mean ulnar deviation increased significantly from 27 degrees (SD 9 degrees) at baseline to 31 degrees (SD 8 degrees) at 12 months ( *p* = 0.013, paired *t* -test). The mean radial deviation remained at the same level, from at 19 degrees (SD 9 degrees) at baseline to 19 degrees (SD 7 degrees) at 12 months ( *p* = 0.227, paired *t* -test). The mean grip strength improved significantly ( *p* ≤ 0.001), from 24.6 kg (SD 12.4) at baseline to 31.9 kg (SD 11.3) at 12 months after surgery.

**Fig. 2:**
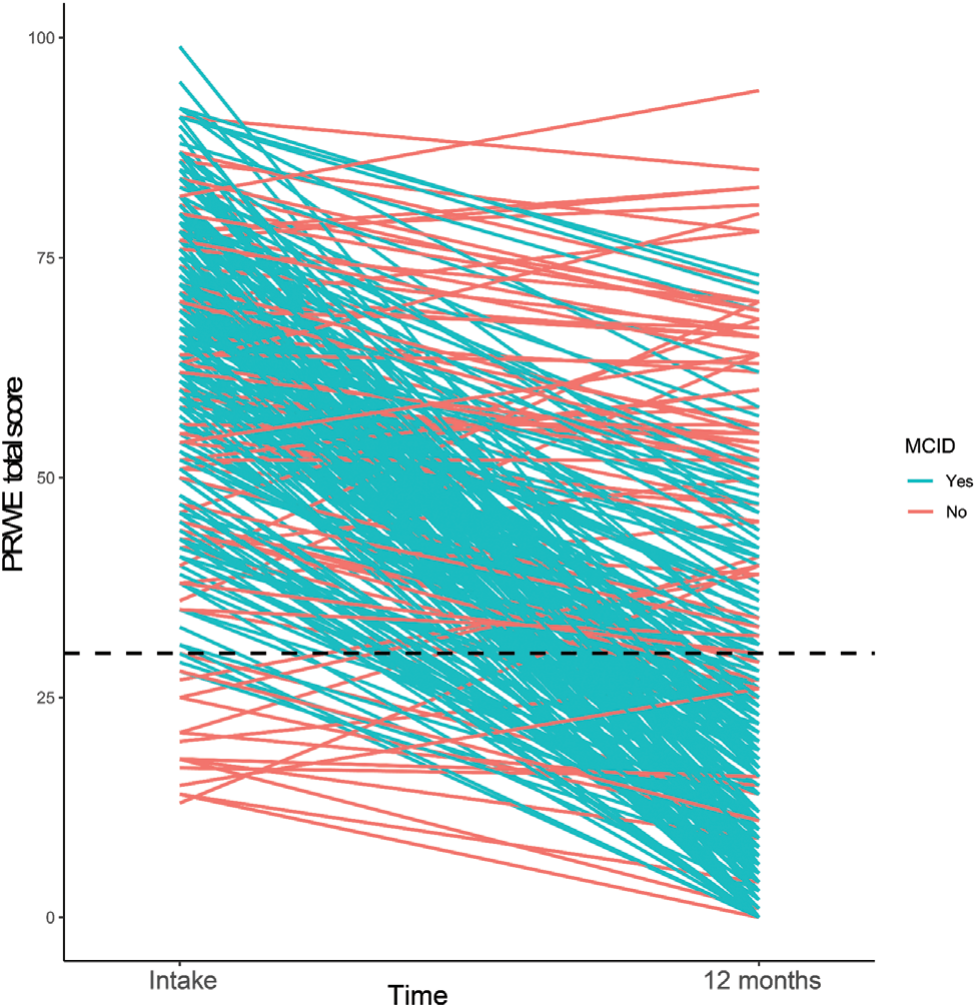
Patient-rated wrist evaluation (PRWE). MCID, minimal clinically important difference.

We compared patient, disease, and surgical characteristics between patients who did and did not reach the MCID. Patients who did not reach the MCID had a longer history of complaints (mean difference = 9 months); otherwise, we found no differences between the two groups in baseline characteristics ( Table 1 ). Fig. 2 shows the changes in PRWE score over time and whether patients reached the MCID. Most patients improved and demonstrated less pain and functional problems after 12 months, but 35% of patients had an improvement of <24 points (MCID). Of patients with a score at baseline <34, only 14% reached the MCID whereas in patients with a score at baseline ≥34, 69% reached the MCID ( Fig. 2 ).

**Table 1:** Patient characteristics

MCID reached	No	Yes	*p* -Value
Number of patients	97	177	
Age, mean (SD)	38 (13)	39 (12)	0.45
Sex, male (%)	33 (34)	45 (25)	0.17
Duration of complaints in mo, mean (SD)	26 (37)	17 (20)	**0.01**
Occupation (%)			
No paid labor	20 (21)	21 (12)	0.14
Light physical labor	28 (29)	68 (38)	
Moderate physical labor	29 (30)	46 26)	
Heavy physical labor	20 (21)	42 (24)	
Second opinion, no (%)	67 (69)	136 (77)	0.81
Previous hand therapy, no (%)	30 (33)	61 (35)	0.76
Dominant side treated, yes (%)	57 (59)	110 (62)	0.68
Ulna plus, yes (%)	83 (86)	158 (89)	0.48
Tear location (%)			
Central	1 (1)	5 (3)	0.65
Dorsal	3 (3)	3 (2)	
Peripheral	44 (46)	93 (53)	
Radial	9 (10)	16 (9)	
None	2 (2)	6 (3)	
Unknown (no arthroscopy)	36 (38)	54 (30)	
Combined Procedures, no (%)	87 (90)	160 (90)	1.00
Complications, no (%)	85 (88)	160 (90)	0.83
PRWE total score at baseline, mean (SD)	53 (22)	67 (14)	< **0.01**

Abbreviations: MCID, minimal clinically important difference; PRWE, patient-rated wrist evaluation; SD, standard deviation.

Note: The bold is to illustrate significant findings *P* <0.05.

**Fig. 3:**
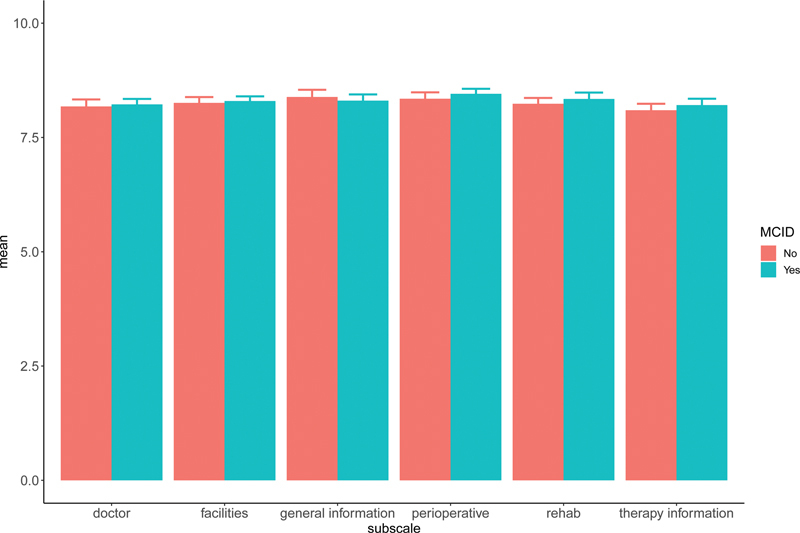
Patient-rated experience measure (PREM).

Fig. 3 shows no difference in the experience with the physician, facilities, preoperative process, rehabilitation, therapy information, and general information between patients who did and did not reach MCID.

**Fig. 4:**
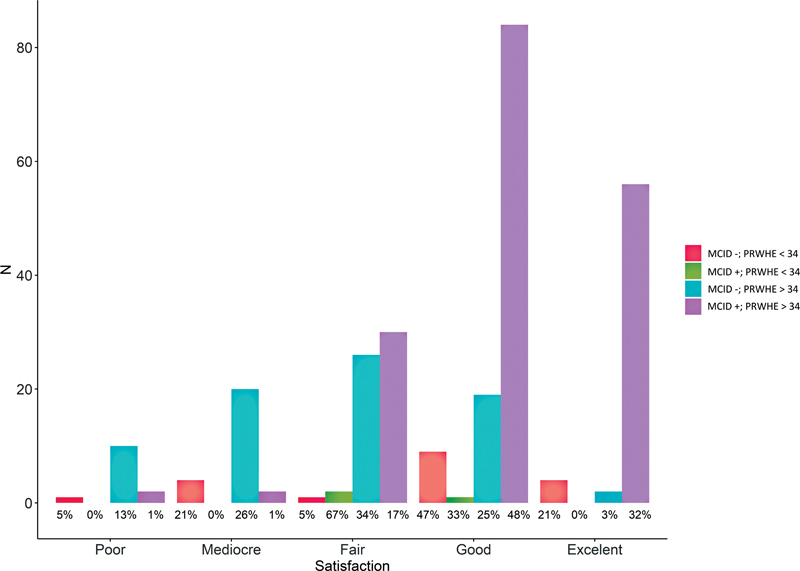
Satisfaction. MCID, minimal clinically important difference.

Fig. 4 demonstrates the distribution of satisfaction with treatment in relation to the PRWE score at baseline and reaching the MCID. A low PRWE score at baseline was associated with poor, fair, or mediocre outcomes. A high PRWE score at baseline was more closely associated with good-to-excellent rated outcomes. Patients ( *n* = 97) who failed to reach the MCID were less satisfied with the result of the surgical procedure ( *p* < 0.001).

## Discussion

We found that a PRWE threshold at baseline <34 often led to dissatisfaction with the outcome, but this did not necessarily lead to dissatisfaction with the treatment experience. Patients who failed to reach the MCID after open reinsertion of the TFCC (35%) were equally satisfied with their treatment experience (i.e., physician communication and facilities) as patients who reached the MCID.

Often, laboratory results are used as a cutoff point for elective surgery. For example, we would not operate on a patient with a low hemoglobin level or prolonged bleeding time. However, hand surgeons are much less acquainted with use of a PROM at baseline in relation to indication setting. We showed the possible predictive value of PROM measurements at baseline in open surgery for the TFCC. In hip arthroplasty, similar findings have been reported by Yeo et al. 20 They found that the hip PROM score at baseline could predict reaching the MCID with a threshold of 24, but it did not correlate with satisfaction with the surgical procedure. There is evidence that a preoperative PROM is useful in predicting reaching the MCID after total knee replacement. 21

Patients who did not reach MCID had a longer history of complaints. This finding is of interest and should be the subject of more research. Is it possible to conclude that we should operate sooner and only allow a certain period for nonoperative treatment? We are unable to answer this question. More research is needed to refine treatment options.

In retrospect, one could wonder why a surgical procedure was advised when only few functional problems and a relatively low pain score on the PRWE were present? Our research showed that a PRWE at baseline <34 in all, but three patients resulted in failure to reach the MCID. We tried to understand why patients with a few functional problems and low pain levels were considered to meet the indication for surgery. However, the joint decision-making at the outpatient clinic was not monitored in sufficient detail in individual patient records to provide an answer. Our institution is a highly specialized clinic, so most patients might expect (and prefer) surgical intervention instead of hand therapy or nonoperative management. In other settings, surgeons and patients might make different treatment choices. We could not find an exact answer to this question in the literature, but our mean PRWE at baseline of 63 was comparable with that of four studies used in a review by Robba et al: 59. 22

Recently, Robba et al 23 concluded that there is little consensus among surgeons on the management of TFCC injuries, so any information on treatment choices might be considered useful. In their study, they mention that labeling a TFCC injury as a “tear” might lead to the patient's perception that the “tear” must be fixed. This perception might explain why patients with relatively low levels of pain and functional loss still chose surgery in our cohort. Conversely, patients may have specific goals for their surgical procedure, such as to solve complaints during specific activities at work or during sporting activities. These specific complaints could go undetected in a standard PRWE questionnaire. These patients may present with, on average, low scores for pain and good scores for function while experiencing sufficiently specific complaints to warrant surgery.

To gain more insight into the specific treatment goals of patients, we recently started to collect data on patient-specific problems using the patient-specific functional scale (PSFS). 24 The PSFS asks patients which task they want to improve with their treatment, and then asks to rate this task on a scale of 0 to 10. The same task is evaluated at follow-up. These data could help us in the future to improve the shared decision process.

Another possible confounder could have been the patient's experience as defined by the PREM questionnaire. We showed that there was no significant difference in experience between our groups ( Fig. 3 ). The group that did not reach the MCID were not, in general, detractors (someone unhappy with your brand, product, or service). They were satisfied with the processes surrounding treatment (i.e., facilities, physician, explanation, therapy rehabilitation, general information). The only answer to the PREM question that differed was the question about satisfaction with the surgical outcome. Not reaching MCID on the PRWE correlated with low satisfaction on the surgical result.

A possible limitation is that we used the question: 'How satisfied are you with the end result?' as an anchor for calculating the MCID. In the anchor-based method for MCID calculation, the lead question asks for a difference in the situation before and after surgery. In 2005, rheumatologists first started using the term “patient-acceptable symptom state” (PASS). 25 26 27 PASS is the value beyond which patients consider themselves to be “well.” It is calculated as the threshold where 75% of patients consider their treatment to be successful. We asked our patients about satisfaction with the end result. Hence, we suggest that the calculated value of 24 points is better described as a clinical discriminator for satisfaction with the end result. The cutoff point that is now calculated defines the minimal points of improvement on a combined scale of patient-reported pain and function for a patient to be satisfied with the end result. We agree with Tubach et al 25 that this discriminator is superior to the traditional MCID, where one searches for the minimal difference in patient-reported outcome that can result in detection by the patient. For example, if a patient had a PRWE at baseline of 90 and improved after surgery by 14 points (MCID as calculated by Sorensen et al 14 ) to 76, this patient may have detected change but could be dissatisfied with the marginally improved end result. Also, we had a generally satisfied patient group and, although we corrected for this, in these situations, anchor-based methods are suboptimal. 16 28 In our opinion, the MIC predict calculation method is the optimal method available for our population. Conversely, a possible “ceiling effect” could prevent patients with a low PRWE at baseline to reach the MCID by default because the PRWE cannot become less than zero. However, we demonstrated that such patients were, overall, dissatisfied with the end result. Therefore, we would advise to reconsider surgery if the PRWE at baseline was <34. We now set a threshold for PRWE at baseline of 34 which allows our surgeons to use this tool to optimize their individualized and patient-centered treatment advise.

The use of PROMS at baseline to guide indications for surgery can be beneficial but has not been accepted widely. We demonstrated that for open TFCC surgery, a PRWE score at baseline <34 was associated with failure to reach the MCID at 12-month follow-up. Also, patients with a PRWE at baseline <34 failed to reach MCID while demonstrating no difference in overall treatment experience (PREM). The PRWE score at baseline can indicate a clinically meaningful outcome in open surgery for the TFCC. We advise reconsidering the indication for surgery with a baseline PRWE score <34.
