# Supplementary material for: Patient-Rated Wrist Evaluation Threshold for Successful Open Surgery of the Triangular Fibrocartilage Complex
Source: J Wrist Surg. 2023 Jul 7;13(4):302–9. doi: 10.1055/s-0043-1771010 (PMC11254475; doi:10.1055/s-0043-1771010)
Supplement: Supplementary file 1 — Supplementary Material [file 10-1055-s-0043-1771010-s2300017.pdf]

Appendix A

Hand–Wrist Study Group collaborators: R.A.M. Blomme, D.J.J.C. van der Avoort, A. Kroeze, J. Smit, J. Debeij, E.T. Walbeehm, G.M. van Couwelaar, G.M. Vermeulen, J.P. de Schipper, J.F.M. Temming, J.H. van Uchelen, H.L. de Boer, K. Harmsen, O.T. Zöphel, T. M. Moojen, X. Smit, R. van Huis, P.Y. Pennehouat, K. Schoneveld, R.M. Wouters, J.J. Veltkamp, A. Fink, W.A. de Ridder, J. Tsehaie, R. Poelstra, M.C. Janssen, P.O. Sun, V.J.M.M. Schrier, L. Hoogendam, J.S. Teunissen, M. ter Stege, H.P. Slijper.

 © 2023. The Author(s).

Supplementary Material S1 PREM questionnaire[a]

**Physician communication and competence**										
What do you think of the personal contact with the doctor?	1	2	3	4	5	6	7	8	9	10	
How well did the doctor listen to what you have to say?	1	2	3	4	5	6	7	8	9	10	
How well did the doctor treat you (i.e., with respect)?	1	2	3	4	5	6	7	8	9	10	
What do you think of the time the doctor has taken for you?	1	2	3	4	5	6	7	8	9	10	
Did the doctor explain things in an understandable way?	1	2	3	4	5	6	7	8	9	10	
What do you think of the medical expertise of the doctor?	1	2	3	4	5	6	7	8	9	10	
**Perioperative care**											
What do you think of the medical expertise of the anesthesiologist?	1	2	3	4	5	6	7	8	9	10	NA
What do you think of the guidance during the operation by the anesthesiologist/anesthesia employee?	1	2	3	4	5	6	7	8	9	10	NA
How do you feel about the type of anesthesia used?	1	2	3	4	5	6	7	8	9	10	NA
What do you think of the guidance/care provided by the nursing staff?	1	2	3	4	5	6	7	8	9	10	NA
**Postoperative care**											
What do you think of the hand therapist's guidance before and after treatment?	1	2	3	4	5	6	7	8	9	10	NA
What do you think of the information that the hand therapist has given to you?	1	2	3	4	5	6	7	8	9	10	NA
What do you think of the alignment in communication between the hand therapist and doctor?	1	2	3	4	5	6	7	8	9	10	NA
What do you think of the aftercare provided by the clinic (recovery period, controls, medication, emergency)?	1	2	3	4	5	6	7	8	9	10	NA
**General information**											
What do you think about the quality of the information brochure?	1	2	3	4	5	6	7	8	9	10	NA
What do you think of the information on the Web site?	1	2	3	4	5	6	7	8	9	10	NA
**Treatment information**											
How adequate was the information provision prior to your treatment?	1	2	3	4	5	6	7	8	9	10	
Are you well informed about the results, alternatives, and risks of treatment?	1	2	3	4	5	6	7	8	9	10	
What do you think of information about the aftercare (checks, emergencies, etc.) after your treatment?	1	2	3	4	5	6	7	8	9	10	NA
**Quality of facility**											
What do you think of the telephone accessibility of the clinic?	1	2	3	4	5	6	7	8	9	10	
How well were you assisted via telephone?	1	2	3	4	5	6	7	8	9	10	
What do you think of the hygiene in the clinic?	1	2	3	4	5	6	7	8	9	10	
What do you think of the clinic's accessibility and parking?	1	2	3	4	5	6	7	8	9	10	
In your opinion, has your clinic performed your treatment safely?	1	2	3	4	5	6	7	8	9	10	NA
How were you received at the clinic (hospitable, friendly, etc.)?	1	2	3	4	5	6	7	8	9	10	

Abbreviations: NA, not applicable; PREM, patient-reported outcome measure.
[a]Questions translated from original, Dutch questionnaire.

 © 2023. The Author(s).

Supplementary Table S1 STROBE checklist of items that should be included in a scientific article

Title and abstract
1. Indicate the study's design with a commonly used term in the title or the abstract
2. Provide in the abstract an informative and balanced summary of what was done and what was found
Introduction
Background/rationale: Explain the scientific background and rationale for the investigation being reported
Objectives: State specific objectives, including any prespecified hypotheses
Methods
Study design; Present key elements of study design early in the article
Setting: Describe the setting, locations, and relevant dates, including periods of recruitment, exposure, follow-up, and data collection
1. Give the eligibility criteria, and the sources and methods of case ascertainment and control selection
2. Give the rationale for the choice of cases and controls
Participants: For matched studies, give matching criteria and the number of controls per case
Variables: Clearly define all outcomes, exposures, predictors, potential confounders, and effect modifiers. Give diagnostic criteria if applicable
Data sources/measurement. For each variable of interest, give sources of data and details of methods of assessment (measurement). Describe comparability of assessment methods if there is more than one group
Bias: Describe any efforts to address potential sources of bias
Study size: Explain how the study size was arrived at
Quantitative variables: Explain how quantitative variables were handled in the analyses; if applicable, describe which groupings were chosen and why
Statistical methods
1. Describe all statistical methods, including those used to control for confounding
2. Describe any methods used to examine subgroups and interactions
3. Explain how missing data were addressed
4. If applicable, explain how matching of cases and controls was addressed
5. Describe any sensitivity analyses
Results
Participants
1. Report numbers of individuals at each stage of study, e.g., numbers potentially eligible, examined for eligibility, confirmed eligible, included in the study, completing follow-up, and analyzed
2. Give reasons for nonparticipation at each stage
3. Consider use of a flow diagram
Descriptive data
1. Give characteristics of study participants (e.g., demographic, clinical, social) and information on exposures and potential confounders
2. Indicate number of participants with missing data for each variable of interest *Outcome data* report numbers in each exposure category or summary measures of exposure
Main results
1. Give unadjusted estimates and, if applicable, confounder-adjusted estimates and their precision (e.g., 95% confidence interval); make clear which confounders were adjusted and why they were included
2. Report category boundaries when continuous variables were categorized
3. If relevant, consider translating estimates of relative risk into absolute risk for a meaningful time period
Other analyses
Report other analyses done, e.g., analyses of subgroups, interactions, and sensitivity analyses
Discussion
Key results: Summarize key results with reference to study objectives

(*Continued*)

 © 2023. The Author(s).

Supplementary Table S1 (*Continued*)

Limitations: Discuss limitations of the study, taking into account sources of potential bias or imprecision; discuss both direction and magnitude of any potential bias
Interpretation: Give a cautious overall interpretation of results that considers objectives, limitations, multiplicity of analyses, results from similar studies, and other relevant evidence
Generalizability: Discuss the generalizability (external validity) of the study results
Other information
Funding: Give the source of funding and the role of the funders for the present study and, if applicable, for the original study on which the present article is based

 © 2023. The Author(s).
